# Maternal hsa-miR-423-5p associated with the cognitive development of babies in pregnant women without mental disorders

**DOI:** 10.3389/fnhum.2024.1322820

**Published:** 2024-02-29

**Authors:** Cainá Corrêa do Amaral, Fernanda Nedel, Camila Perelló Ferrúa, Tiago Fernandez Garcia, Geovanna Peter Corrêa, Roberta Giorgi, Aline Longoni dos Santos, Adriano Martimbianco de Assis, Luciana de Avila Quevedo, Gabriele Cordenonzi Ghisleni, Mariana Bonati de Matos, Karen Amaral Tavares Pinheiro, Jéssica Puchalski Trettim, Ricardo Tavares Pinheiro

**Affiliations:** ^1^Post-Graduate Program in Health and Behavior, Catholic University of Pelotas, Pelotas, Brazil; ^2^Anatomy Department, Federal University of Pelotas, Pelotas, Brazil; ^3^Department of Ophthalmology, Faculty of Medicine, Federal University of Rio Grande, Rio Grande, Brazil

**Keywords:** microRNA, pregnancy, babies, cognitive development, retinoic acid

## Abstract

**Background:**

MicroRNAs (miRNAs) are small non-coding RNAs capable of regulating gene expression post-transcriptionally. MiRNAs are recognized as key regulators of diverse biological and developmental processes. During the pregnancy–puerperal cycle, numerous changes occur in the female body for the formation, growth, and development of the baby. After birth, there is a critical period in child development, as rapid gains in the physical, cognitive, and socio-emotional domains constitute the “building blocks” of children’s later growth.

**Objective:**

The aim of this study was to investigate the association between maternal expression of hsa-miR-423-5p during the first and second trimesters of pregnancy and neurocognitive development at 90 days of life in infants. Methods: This is a longitudinal study included in a population-based cohort study, carried out in a city in southern Brazil. The Bayley III was used to assess the babies’ cognitive development. Blood samples from mothers were obtained for RNA extraction from serum and analysis of miRNA expression by qRT-PCR.

**Results:**

In total, 87 dyads (mother–baby) were included. The average gestational age was 15.86 weeks (SD ± 5.55). An association of maternal miRNA with infant cognitive development was found; as maternal miR-423-5p increases, infants’ cognitive development increases by 2.40 (95% CI 0.37; 4.43, *p* = 0.021) points at 3 months of age.

**Conclusion:**

In this context, it is suggested to use this miRNA as a biomarker of child neurocognitive development detectable in the prenatal period, thus allowing the planning of early interventions.

## 1 Introduction

MicroRNAs (miRNAs) are highly conserved short 18–25-nucleotide non-coding RNAs that bind and post-transcriptionally regulate messenger RNA (mRNA). MiRNAs regulate mRNA by binding to a short core sequence in the 3′ untranslated region (UTR) of mRNA or non-coding RNAs (Tay et al., 2011) and lead to inhibition of translation or target mRNA degradation (Simonson and Das, 2015; Vishnoi and Rani, 2017). Importantly, miRNAs can target epigenetic regulators, which play a role in fetal metabolic programming (Sookoian et al., 2013), mediating long-term effects on target cells and developing organs and tissues. Epigenetic factors and miRNAs are reciprocally regulated (Pasquinelli, 2012; Floris et al., 2015). Thus, miRNAs are recognized as key regulators of diverse biological and developmental processes in eukaryotes such as cell proliferation and differentiation, maintenance of tissue identity, apoptosis, and immune system development (Floris et al., 2016).

Based on this, attention is drawn to the pregnancy–puerperal cycle, a period of numerous changes in the female organism and several processes triggered for the formation of the fetus, in addition to the growth and development of the baby. The biological dialogue between a mother and her offspring starts from the implantation of embryo in the uterus and continues during fetal life via the maternal–placental–fetal axis (Power and Schulkin, 2013). In this period, the expression of miRNAs is ubiquitous; they are present in cells and body fluids of both maternal and fetal origin (Floris et al., 2016).

After birth, there is a critical period in child development, as rapid gains in physical, cognitive, and socio-emotional domains constitute the “building blocks” of children’s later growth. The multiple domains of child development are interconnected. For example, good nutrition during the early years is essential for healthy physical development and enhances cognitive and socio-emotional growth (Bornstein et al., 2012). Responsiveness in the parent–child relationship promotes healthy socio-emotional development and improves physical and cognitive outcomes. Thus, infant and child development depends on the significant interdependence of developmental streams (Wilks et al., 2010).

Cognitive development is considered the foundation of intelligence. Assessment of infant and toddler intelligence depends on progression in two developmental domains: problem solving and language. Children advance in these domains by learning. Learning requires the ability to direct and sustain attention as well as the ability to manipulate information. Key aspects of cognitive development include memory, representational competence, attention, and processing speed. Successful cognitive development requires progress in all these domains (Wilks et al., 2010).

In view of all this, the epigenetic role of miRNAs in the gestational period with fetal interaction is observed. Therefore, in this study, we investigated the association between has-miR-423-5p expression in mentally healthy pregnant women during the first and second trimesters of pregnancy and neurocognitive development at 90 days of life in babies to identify the possible impacts of changes in maternal miRNAs on the development of their children.

## 2 Materials and methods

### 2.1 Design

This is a longitudinal study included in a population-based cohort study, carried out in a city in southern Brazil. The research ethics committee of the Catholic University of Pelotas, under opinion number 1,729,653, approved the cohort project to which it is linked. For more details about sample capture, read the publications of Pinheiro et al. (2021, 2022). The study design is similar to the one carried out by Nedel et al. (2023), in which dyads (mother–baby) were included. However, we had the exclusion criteria as a difference, which were mood disorders anxiety disorders, or suicidal ideation until the moment of evaluation and collection of biological material. Therefore, 60 days after the initial assessment, all women were re-evaluated in relation to the presence of mental health disorders, thus ensuring that these pregnant women did not present the aforementioned disorders during this period, which differentiates our sample from the study carried out by Nedel et al. (2023), which did not exclude the manifestation of mental health disorders at any time during the evaluation periods.

### 2.2 Instruments

The Bayley Scale of Infant and Toddler Development III (Bayley-III) was used to evaluate the cognitive development outcome of babies 90 days after birth through composite scores. The socioeconomic evaluation of the participants was carried out using the ABEP classification, with the levels categorized as follows: A + B (high levels), C (medium levels), and D + E (low levels).^1^ Mental disorders were assessed using the Mini International Neuropsychiatric Interview (Mini Plus 5.0.0 Brazilian Version).

Maternal variables, such as age and education (collected in complete years and subsequently categorized into tertiles), gestational age in weeks, first pregnancy (yes/no), and planned pregnancy (yes/no), were collected using questions from a general structured questionnaire. The body mass index (BMI) was assessed by weight/height2 (kg/m^2^), with weight measured using an anthropometric scale and height using a stadiometer. The prematurity variables were collected from the baby’s medical records, and the mother answered questions about breastfeeding.

### 2.3 Blood sample collection and processing

Blood samples were obtained by venipuncture (10 mL) from all pregnant women. The serum blood was immediately centrifuged at 3,000 g for 10 min at 4°C, and the supernatant was transferred to RNase-/DNase-free tubes and stored at −80°C until RNA extraction.

### 2.4 RNA extraction and qRT-PCR analysis

Total RNA was extracted from serum using a mirVana PARIS kit (Thermo Fisher Scientific) according to the manufacturer’s protocol. The RNA was reverse transcribed to cDNA using TaqMan miRNA Assays (Thermo Fisher Scientific) according to the manufacturer’s protocol. Quantitative PCR was performed using a TaqMan Fast Advanced Master Mix kit (Thermo Fisher Scientific). Thermal cycling was conducted according to the recommended program of the manufacturer; in all experiments, a negative control was performed and the samples were duplicated. The TaqMan miRNA Assays used in this study and their TaqMan Assay IDs are as follows: miR-423-5p (478090_mir/nucleotide sequence UGAGGGGCAGAGAGCGAGACUUU). The CT values were normalized using the delta CT method with the endogenous controls (miR-17-5p/nucleotide sequence CAAAGUGCUUACAGUGCAGGUAG). To determine the corresponding ΔCT value, the CT value of the target gene miRNAs was subtracted from the miR-17–5p CT value.

### 2.5 Statistical analysis

Data analysis to investigate the association of gestational levels of hsa-miR-423-5p with the outcome of child cognitive performance at 90 days was performed with crude linear regression and subsequently adjusted (backward method) for maternal age, gestational weeks, primiparity, planned pregnancy, socioeconomic level, maternal education, maternal gestational BMI, prematurity, and exclusive breastfeeding. The significance level considered was 0.05.

## 3 Results

We analyzed data from 87 dyads (mother–infant) with an assessment of the infants 90 days postpartum. The mean gestational age within the sample was 15.86 weeks (SD ± 5.55); the most prevalent socioeconomic level was C (66.7%), followed by A + B (20.7%) and D + E (12.6%). Mean maternal age was 27.66 years (SD ± 5.40), and maternal schooling was 10.4 completed years of study (SD ± 3.34). Furthermore, 58.6% of pregnant women were multiparous, and 72.4% planned the pregnancy. The average maternal BMI among pregnant women was 27.87 kg/m^2^ (SD ± 5.50). Regarding babies, 89.7% were born at term and 52.9% were exclusively breastfed at the time of the interview. The overall mean of has-miR-423-5p was −0.06 (SD ± 1.6), and the composite cognitive development score of Bayley-III was 101.09 (SD ± 11.80; Table 1).

**Table 1:** Sample characterization.

Variables	N/Mean	%/SD
Age		
Up to 23 years	16	18.4
Between 24 and 29 years old	37	42.5
30 years or older	34	39.1
Previous pregnancy		
No	36	41.4
Yes	51	58.6
Planned pregnancy		
No	24	27.6
Yes	63	72.4
Socioeconomic class		
A and B	18	20.7
C	58	66.7
D and E	11	12.6
Schooling		
Elementary school (up to 8 years of study)	19	21.8
High school (between 9 and 11 years of study)	44	50.6
University education (12 years of study or more)	24	27.6
Prematurity*		
No	78	89.7
Yes	7	8.0
Exclusive breastfeeding*		
No	39	44.8
Yes	46	52.9
Gestational age	15.86	5.55
Body mass index	27.87	5.50
hsa-miR-423-5p	−0.06	1.36
Cognitive development score	101.09	11.80

*Variables with missing.

**Figure 1:**
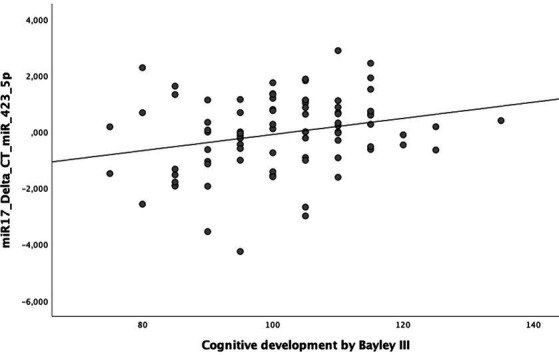
Scatter plot correlating the Bayley-III composite cognitive development score and hsa-miR-423-5p, *p* = 0.021.

In the scatter plot, Figure 1 shows a significant correlation between has-miR-423-5p and the cognitive development score (*p* = 0.021). Collinearity diagnoses demonstrate tolerance >0.1 and VIF <10, not indicating multicollinearity among any of the tested variables.

Linear regression for the outcome of childhood neurocognitive development shows the association between miR-423-5p and the domain of cognitive development. As maternal miR-423-5p increases, it increases by 2.26 (CI 95% 0.36; 4.16, *p* = 0.020) points to the average cognitive development of babies at 3 months of age. When analyzing the adjusted regression model, it also showed an association with cognitive development even after removing confounding variables (age, BMI, previous pregnancy, planned pregnancy, socioeconomic class, schooling, prematurity, exclusive breastfeeding, and gestational age). As maternal miR-423-5p increases, the average cognitive development of babies increases by 2.40 (CI95% 0.37; 4.43, *p* = 0.021) points at 3 months of age, data shown in Table 2.

**Table 2:** Multivariate analysis according to the cognitive development score of babies at 90 days of life.

Variables	Crude analysis		Adjusted analysis	
	RR (CI 95%)	*p*-value	RR (CI 95%)	*p*-value
Age (up to 23 years*)	1.84 (−1.60;5.29)	0.290	0.27 (−4.02;4.56)	0.900
Body mass index	−0.16 (−0.62;0.30)	0.499	−0.20 (−0.68;0.27)	0.398
Previous pregnancy (no*)	−1.22 (−6.35;3.91)	0.638	−0.47 (−6.90;5.95)	0.882
Planned pregnancy (no*)	−2.08 (−7.73;3.56)	0.465	−1.77 (−7.90;4.37)	0.568
Socioeconomic class (class A and B*)	−1.31 (−5.73;3.10)	0.556	0.83 (−4.69;6.35)	0.764
Schooling (elementary school*)	2.56 (−1.00;6.13)	0.157	2.59 (−1.09;6.28)	0.165
Prematurity (no*)	−8.16 (−17.41;1.09)	0.083	−5.77 (−15.78;4.24)	0.255
Exclusive breastfeeding (no*)	1.92 (−3.21;7.04)	0.459	2.47 (−2.86;7.80)	0.359
Gestational age	−0.15 (−0.61;0.31)	0.511	−0.10 (−0.60;0.40)	0.700
hsa-miR-423-5p	**2.26 (0.36;4.16)**	0.020*	**2.40 (0.37;4.43)**	0.021*

Bold values provided represent data with statistically significant results. The *symbol provided demonstrates the missing variables.

## 4 Discussion

This study investigated the influence of maternal miRNA expression on the cognitive development of 90-day-old infants and confirmed the hypothesis that hsa-miR423-5p in mentally healthy pregnant women is associated with the cognitive development scores of infants at 3 months of age. It is important to highlight that these results are independent of maternal mental disorders, as this was an exclusion criterion for this study. In this study, we chose to select mothers without any previous diagnosis of mental disorders and thus exclusively report changes in the gestational period and the impacts on the cognitive development of babies, which differentiates us from studies published to date.

The contribution of this study is the identification of miR-423-5p expression levels in maternal blood and its ability to influence the cognitive development of infants at 90 days of age, which suggests a probable biomarker to be used in future. In addition, we call attention to the study previously published by our research group (Nedel et al., 2023), which showed that the expression of specific microRNAs in the maternal blood during pregnancy would be associated with the cognitive development of babies; however, it was also evident that the presence of mood disorders during pregnancy was also associated with child development deficit in this period. A question of reverse causality and another question of possible mediation of the child development outcome was posed. How to answer whether it is gestational depression that alters the expression of microRNAs or vice versa? Would the alteration in infant development manifest itself without the presence of mood disorders and anxiety during pregnancy? Thus, when it was impossible to answer the first question, which is a limitation of the study, we selected mothers without mental disorders in order to reduce the influence of depression and gestational anxiety as confounders of cognitive development. The answer to that question is that we present.

The association highlighted the influence of maternal miRNA on the fetus, which can be justified in two ways, direct or indirect exposure. In direct exposure, miRNAs pass directly from mother to fetus through the placenta, influencing the baby’s neurodevelopment. However, this association can also be perceived through indirect exposure, in which miRNAs affect signaling pathways, which control the baby’s development. Both situations are viable, as the literature has increasingly reinforced the developmental origins of health and disease (DOHaD) hypothesis, which postulates that the perinatal environment can impact fetal health and adult life. According to a literature review by Lapehn and Paquette (2022), including 28 articles published between 2016 and 2021, maternal exposure is linked to health outcomes such as birth weight, fetal growth, or infant neurobehavior (Lapehn and Paquette, 2022). Among the justification for studies to modulate the fetus are microRNAs that are expressed in the placenta and are involved in fetal and maternal signaling through circulation in the maternal circulation within extracellular vesicles known as exosomes (Mitchell et al., 2015). The identification of placental-specific miRNAs secreted into maternal blood from exosomes is an area of growing interest as a non-invasive biomarker for pregnancy health (Mouillet et al., 2015), as it is analyzed from peripheral venous blood collection, as performed in this study. Most epigenetic methods of genetic control are studied in the placenta; however, its application to specific prenatal exposures and postnatal health outcomes is a growing area of research, given the relevance of the topic.

To further resource our findings, both forms of maternal miRNA influence on the infant can affect embryology and thus lead to outcomes in fetal brain development. Our sample was evaluated for serum levels of hsa-miR423-5p at approximately 15 weeks of gestation, on average. During this period, the rate of neurogenesis and neuronal migration is accelerated, as relatively small perturbations can significantly alter the structure and function of the maturing brain, as demonstrated in the study by Zhan et al. (2013), which created a time-spatial atlas of human fetal brain development in the second trimesters of pregnancy (Zhan et al., 2013).

To talk about the role of miR-423-5p in brain development, we found the study by Mao et al. (2019), which stated a direct interaction of miR-423-5p, miR-485-5p, and miR-666-3p with expression of cytochrome P450, family 26, subfamily B, polypeptide 1 (Cyp26b1). The authors performed a Western blot assay that revealed that miR-423-5p, miR-485-5p, and miR-666-3p reduced the protein level of Cyp26b1 in Schwann cells in cell culture, demonstrating that these miRNAs can regulate the expression of Cyp26b in these cells (Mao et al., 2019).

With these data, it is possible to hypothesize a pathway between maternal miRNA expression and the baby’s cognitive development at 3 months of age. Our results demonstrate a positive association between maternal miRNA and the baby’s development score, so it is possible to infer that the increase in miR-423-5p found in this study is capable of regular levels of Cyp26b1 levels, as found by Mao et al. (2019). From this, it is seen in the literature that cytochrome is capable of controlling the diffusion of retinoic acid (RA) in our cells; that is, the activity of Cyp26b1 regulates the levels of RA (Ross and Zolfaghari, 2011). Continuing our hypothesis, it is possible that the increase in maternal miR-423-5p regulates the levels of Cyp26b1 and, consequently, the levels of RA.

Based on this, it is worth highlighting that RA reaches the human body through the intake of vitamin A, which is essential for embryonic development (Ghyselinck and Duester, 2019). AR plays a fundamental role in the formation of the embryo since the period of organogenesis. Vitamin A deficiency during pregnancy can result in a set of defects and malformations in the fetus, referred to as vitamin A deficiency syndrome (See et al., 2008). On the other hand, excessive intake of vitamin A, AR, or acidic retinoid analogs is highly teratogenic and induces abnormalities that are not very different from deficiency, including defects in the craniofacial, central nervous, and cardiovascular systems (Ross and Zolfaghari, 2011).

In this sense, it should be noted that a relationship between RA and brain development has already been established. The literature suggests that AR plays a crucial role in neurogenesis (Bonnet et al., 2008; Park et al., 2016; Dutta et al., 2023), as well as in synaptic plasticity, including in adult patients (Lenz et al., 2021). The physiological actions of RA begin early in development and continue throughout life. In this sense, it was demonstrated by Shibata et al. (2021) that AR signaling is required for proper gene expression in the prefrontal cortex, spinogenesis, and long-range connectivity (Shibata et al., 2021).

Furthermore, the relationship among vitamin A, brain development, and cognitive development is evident. An *in vivo* study performed by Jiang et al. (2012) demonstrates that vitamin A deficiency is capable of impairing postnatal cognitive function, causing learning and spatial memory deficits in rats (Jiang et al., 2012). Lai et al. (2018) reported that vitamin A deficiency induces autism-like behaviors in rats (Lai et al., 2018). Both studies show us the important role of vitamin A and RA in cognitive development, especially concerning the gestational period and maternal lifestyle about the consequences and legacies passed on to the fetus, which can remain throughout the life of the mother child, especially when it comes to maternal nutrition, as it is already seen in the literature how much it impacts the baby’s neurodevelopment (Nyaradi et al., 2013; Cortés-Albornoz et al., 2021; Heland et al., 2022).

Cognitive development is a process that begins very early when the individual is still an embryo and receives the entire genetic, physical, emotional, affective, and biological load from their parents (Kadic and Kurjak, 2018). From birth onwards, and stimuli that caregivers provide to the child, to gestation conditions and general care for the baby and mother are fundamental for good development. As it is such an important topic, it is necessary to know the hereditary and environmental factors that can compromise the growth of fetuses and children, mainly with the aim of preventing and identifying risk situations to provide care as early as possible and avoid future impacts on the health of children.

Finally, we recognize that not having evaluated miRNA expression at other times of pregnancy is a limitation of our study, as well as using a single endogenous miRNA, duplicate reactions and not having tested the suggested hypothesis in the discussion. Regarding methodological considerations, we would like to highlight that we previously carried out a pilot study to evaluate the miRNAs, as at the time of the project there was a great lack of literature evaluating miRNA in serum, especially from pregnant women. At this time, as an initial precaution, we quantified the samples using the Spectrophotometer (Thermo Scientific™ NanoDrop™) to evaluate the purification of the extracted RNA. Regarding endogenous control and duplicate reactions, we did not find standardization in the literature, including studies published in recent years using a single endogenous miRNA for control (Sekovanić et al., 2021; Bayati et al., 2022; Couto et al., 2022; Li et al., 2022; Ding et al., 2023; Higashi et al., 2023). Furthermore, Couto et al. (2022) cite duplicate reactions and the other authors do not cite the number of reactions carried out. In this context, we consider the methodology used in this study appropriate to the current scientific scenario. Furthermore, we draw attention to our result, mainly due to its innovative potential in the child neurocognitive area. We are pioneers on the subject and this allows us to foster this relationship, opening paths for new studies, including more robust methodologies to uncover paths responsible for the association found in this study. In this sense, it is essential to emphasize the need for *in vitro* and *in vivo* studies to clarify the reasons why miR-423-5p is capable of influencing the mother–infant trajectory with direct or indirect effects on cognitive development. Studies are also needed to clarify the mechanism of action of miRNA in the context of neurodevelopment during pregnancy and whether this relationship remains over time, as neuroplasticity at this stage of life may combine differences in neurodevelopment, mitigating the effects observed in babies over 3 months of age.

## 5 Conclusion

In summary, an association between the expression of hsa-miR-423-5p in the peripheral blood of mentally healthy pregnant women during the first and second trimesters of pregnancy and the cognitive development scores of infants at 3 months of age was found. This is a pioneering study on the subject; therefore, it is expected that with more studies this miRNA can be used as a biomarker of child neurocognitive development detectable in the prenatal period, thus allowing the planning of early interventions.

## Data availability statement

The original contributions presented in the study are included in the article/supplementary material, further inquiries can be directed to the corresponding author.

## Ethics statement

The studies involving humans were approved by the research ethics committee of the Catholic University of Pelotas. The studies were conducted in accordance with the local legislation and institutional requirements. Written informed consent for participation in this study was provided by the participants’ legal guardians/next of kin.

## Author contributions

CA: Conceptualization, Data curation, Formal analysis, Investigation, Methodology, Validation, Visualization, Writing – original draft. FN: Conceptualization, Data curation, Formal analysis, Investigation, Methodology, Supervision, Validation, Writing – review & editing. CF: Conceptualization, Investigation, Methodology, Writing – original draft. TG: Conceptualization, Investigation, Methodology, Writing – original draft. GC: Conceptualization, Investigation, Methodology, Writing – original draft. RG: Conceptualization, Investigation, Methodology, Writing – original draft. AL: Writing – review & editing. AA: Supervision, Writing – review & editing. LA: Supervision, Writing – review & editing. GG: Supervision, Writing – review & editing. MM: Supervision, Writing – review & editing. KP: Supervision, Writing – review & editing. JT: Conceptualization, Data curation, Formal analysis, Investigation, Supervision, Validation, Writing – review & editing. RP: Formal analysis, Funding acquisition, Investigation, Project administration, Resources, Supervision, Writing – review & editing.

## Footnotes

^1^ www.abep.org
